# Sleep medicine, sleep research, and sleep education: a whole life devoted to sleep

**DOI:** 10.1093/sleepadvances/zpae029

**Published:** 2024-06-05

**Authors:** Michel Billiard

**Affiliations:** Department of Neurology, Gui de Chauliac Hospital, Montpellier, France

**Keywords:** sleep medicine, sleep research, sleep education, sleep societies, European Commission

## Abstract

This article describes my participation in sleep medicine, sleep research, and sleep education, mainly in Europe, between the years 1970 and 2000.

Sleep medicine is a relatively new field of medicine mainly launched by William C. Dement and Christian Guilleminault, at the Stanford Sleep Clinic, California in the seventies. This article enlights my personal itinerary before coming to sleep medicine, provides an overview of the development of sleep medicine in Europe and Montpellier, outlines my own sleep research, and summarizes my role in sleep education.

## A Non-conventional Three-Stage Itinerary Before Coming to the Sleep Field

### Catholic Faculty of Theology at the University of Strasbourg,

I was 18 when I entered the Catholic Faculty of Theology, University of Strasbourg, France, the only French public University delivering recognized higher education diplomas in theology. It was a fascinating experience in the fields of systematic theology, biblical sciences, historical sciences, philosophy, ethic, ancient languages including Latin, Greek, and Hebrew. Unfortunately at that time, France was engaged in the Algerian War, an independence conflict between France and one of its ancient colonies. Thus, at the age of 21, in 1958, I was called up to the Army and forced to put my theological studies in brackets.

### Army, in France and Algeria

I was a simple soldier for 4 months at Noyon in the north of France, an apprentice officer for 8 months at the prestigious Armored-Cavalry Application School of Saumur and finally, a second lieutenant for 16 months in the 25th regiment of dragoons in the Ouarsenis mountains, North Algeria. During that time I had the chance to discover the Arabic population and learn the Algerian language, but I had to face critical engagements with rebels, hence a decoration of the Algerian War Cross before returning to civilian life in France end of 1960.

### Medical schools in Paris and Montpellier

Eventually, after these two experiences, I entered the school of medicine in Paris at 25, passed the Paris and Montpellier internship competitions and decided to quit Paris for Montpellier, the oldest school of medicine in France and a quite attractive city. After 2 years as an intern in the departments of internal medicine, neuro-surgery, neurology, and psychiatry, I chose to complete my internship in the Department of Pathophysiology of Nervous Diseases, held by Pierre Passouant, in the newly opened Gui de Chauliac Hospital, Montpellier. This department included a 28-bed neurology ward, EEG and EMG units, and a two-room sleep unit, the very first one opened in France. Pierre Passouant studied medicine in Montpellier and passed the internship competition in Montpellier in November 1937. He had had his internship interrupted several times by his military service, first as an auxiliary physician in the Barcares camp, where Spanish republican refugees were interned, second as the chief physician of the Bergues Hospital during the battle of Dunkerque, at the end of which he was made prisoner for 10 months in Belgium, and third as a neurologist in the military hospital Val de Grâce in Paris, where he participated in the early development of electroencephalography. Back in Montpellier in 1946, he was rapidly in charge of the electroencephalography and electromyography units. In 1951 he went to the United States where he spent a 6 months period in the Department of Electrophysiology and Neuropsychiatry of the Massachusetts General Hospital in Boston. There, he worked on myasthenia and became acquainted with stereotaxy in the cat. Two years later he opened a laboratory of experimental medicine at the University of Montpellier and initiated studies on the hippocampus and later on the cerebellum, the rhinencephalon, and the bulbar olive in the cat. As soon as 1960 he became interested in sleep and was the first French neurologist to write articles in this field. In the year 1961, he started recording epileptic and narcoleptic patients at night. 10 years later, in 1971, he succeeded in opening a Department of Physiopathology of Nervous Diseases at the newly built Gui de Chauliac Hospital.

## Sleep Medicine

After 2 years as an intern in this department, I became a resident in charge of both the neurology ward and the sleep unit. Two years later, in 1973, I completed my training in sleep medicine in foreign institutions. First, in the Department of Psychological Medicine held by Ian Oswald in the Royal Edinburgh Hospital, Edinburgh, Scotland. Ian’s main interest was the pharmacology of sleep. I learned a lot from him and his assistant Vlasta Brezinova. From then on, we remained in close contact, and in 1989 I had the honor to give a lecture at the famous Sandoz Foundation in Edinburgh. A few months later, I left again Montpellier and spent 14 months as a research fellow in the Sleep Research Center founded and held by William C Dement in the Department of Psychiatry and Behavioral Sciences, School of Medicine, Stanford University. Bill, as we familiarly called him, was recognized as « the father of sleep medicine ». He had been the first researcher to be interested in the connection between rapid eye movements and dreaming, the first to use sleep deprivation as a research tool to unveil various aspects of sleep function per se, the author of the classification and naming of sleep stages in 1957, and the initiator, together with Mary Carskadon, of the multiple sleep latency test used to measure sleepiness. In charge of running the sleep clinic was Christian Guilleminault, a French doctor who had arrived from the hospital La Salpétrière in Paris, in January 1972. Christian was a hard worker and a brilliant lecturer. He was extremely demanding and I’d like to pay a large tribute to him for pushing me in sleep research and sleep education. He was the first to describe the obstructive sleep apnea syndrome and then, together with Riccardo Stoohs, the upper airway resistance syndrome. Staying at Stanford was a great time for me, learning a lot from William C Dement, Christian Guilleminault, Vincent Zarcone, Mary Carskadon, and others. I saw a lot of patients with narcolepsy, idiopathic hypersomnia, and Kleine-Levin syndrome. I interacted with sleep technologists. I met a lot of sleep leaders at Stanford and in different symposia and congresses in the United States. I experienced research mainly in the field of narcolepsy. One of my closest friends in the sleep clinic was Jacques Montplaisir, an MD, PhD, the future director of the Department of Psychiatry, University of Montreal, and Center for Advanced Research in Sleep Medicine, Hôpital du Sacré-Cœur, Montreal. Pierre Passouant, Ian Oswald, William C. Dement and Christian Guilleminault were definitely my first mentors in the field of sleep, but there were others, later in my professional life: Bedrich Roth, the father of idiopathic hypersomnia, Department of Neurology, Charles University, Medical Faculty, Prague, Czechoslovakia; Yukata Honda, the discoverer of the association of HLA-DR2 with narcolepsy, Seiwa Hospital, Neuropsychiatric Research Institute, Tokyo, Japan; and Roger Broughton, who introduced me to chronobiological, behavioral and medical aspects of napping, Division of Neurology, University of Ottawa and Ottawa General Hospital, Canada. I visited them several times in Prague, Tokyo, and Ottawa and regularly communicated with them.

When I returned to Montpellier, at the end of 1974, a few precursors (Appendix Table 1) were interested in sleep disorders. However, sleep medicine was not yet established in Europe. It is in this context that the « Diagnostic Classification of Sleep and Arousal Disorders » was prepared in the years 1977–1978, by a North American dedicated committee, including a chairman, Howard P. Roffwarg, eight members, Robert W. Clark, Christian Guilleminaut, Peter J. Hauri, David J. Kupfer, Laughton E. Miles, Helmut S. Schmidt, Vincent P. Zarcone, Frank J. Zorick, and 2 consultants to the committee, William C. Dement and Allan Rechtschaffen. This classification was published in the journal Sleep in 1979 [1]. It included four sections, section A, disorders of initiating and maintaining sleep (insomnias), section B, Disorders of excessive somnolence, section C, disorders of the Sleep–Wake schedule and section D, Dysfunctions associated with sleep, sleep stages or partial arousals (parasomnias). This first classification led to the progressive opening of sleep disorder centers controlled by well-trained sleep physicians and sleep technologists. They included an outpatient clinic, well-equipped recording rooms, a controlled room, and rooms for performance and cognitive tests. Disorders investigated in these centers included sleep and wake disorders, medical disorders associated with sleep (sleep-related breathing disorders, cardiovascular disorders occurring during sleep, night epilepsies, infants near-miss for sudden infant death syndrome, sleep-related painful erections), and some medical disorders benefiting by polysomnography (mood disorders, neurodegenerative disorders, epilepsies or dysautonomias). As concerns, the Montpellier Sleep Disorders Center, it had the advantage of two well-equipped rooms and one control room created by Pierre Passouant, but it suffered from a rather limited staff. Thus, the priority was to recruit clinicians and researchers and to hire and train sleep technologists. This took time and effort, but within a few years a full staff including physicians, Bertrand Carlander a distinct neurologist, Basile Ondzé an internist interested in sleep, and later Yves Dauvilliers both a neurologist and a physiologist, and researchers, Alain Besset, an INSERM (Institut national de la santé et de la recherche médicale) psychologist, and Mehdi Tafti, a brilliant PhD student. In addition, we had foreign doctors coming for 1 to 2 years to train in sleep medicine and PhD students. Besides, we had well-known researchers coming for a 9-month sabbatical. Meantime, the number of sleep technologists regularly increased. They received patients, completed their medical history, hooked them up, observed them day and night, and interpreted polygraphic recordings. All of them were dedicated to their work and I enjoyed visiting them at night, once or twice a week, to foster their interest in sleep disorders.

## Sleep Research

Here are comments about some of our research at the Wake and Sleep Disorders Center, Gui de Chauliac Hospital, Montpellier, France.

### Epilepsy

My first contact with sleep research was through epilepsy. As a matter of fact, Pierre Passouant and his coworkers Michel Baldy-Moulinier and Marion Delange, had been interested for years in showing the interplay between sleep and epilepsy. Indeed, sleep has an effect on most forms of epilepsy, in modifying the frequency and expression of seizures and of spikes and waves. Reciprocally, seizures either generalized or partial modify the course of sleep. Among generalized idiopathic epilepsies, *tonic-clonic seizures* can be observed during sleep with two frequency peaks, 1 to 2 hours after the onset of sleep and the other one at the end of the night, between 5 and 6 am. These occur during NREM sleep only, most often during stage 2. The seizure momentarily interrupts the course of sleep, which later resumes with little or no REM sleep. The clinical expression of *petit mal absences* can only be witnessed during wakefulness. *Myoclonic seizures of adolescence* occur chiefly within a few minutes to one hour after awakening. As regards partial idiopathic epilepsies, *seizures of frontal origin* occur mostly during sleep, are typically of short duration, and occur in clusters, mainly in the first part of sleep, whereas in *temporal lobe epilepsy*, seizures occur mostly during wakefulness. However, sleep modulates these seizures too. Complex partial seizures starting from sleep evolve to secondary generalized seizures twice as often as seizures starting from wakefulness. My own contribution concerned epilepsies and the sleep–wake cycle [2]. In a population of 77 patients with tonic-clonic seizures, 36.3% of patients had daytime seizures, 28.5% nighttime seizures, 18.1% daytime and nighttime seizures, and 16.8% seizures on awakenings, while in a population of 32 patients with myoclonic seizures of adolescence, 81.2% of patients had seizures on awakening, against 6.2% during nighttime, 6.2% during daytime, and 6.2% during day time and nighttime. As concerns partial seizures, the distinction between frontal lobes and temporal lobes was not yet available at that time. In a population of 127 patients with partial seizures with complex symptomatology, 61.4% of patients had daytime seizures, 29.1% daytime and nighttime seizures, and 9.4% nighttime seizures.

## Disorders of Central Hypersomnolence

### Narcolepsy

#### Competition between REM sleep and NREM sleep.

Narcolepsy with cataplexy is characterized b the occurrence of sleep-onset REM periods. However, narcoleptic patients may present sleep-onset NREM periods as well, so narcolepsy goes with a variable ratio of sleep-onset REM periods to sleep-onset NREM periods. To help clarify this puzzling situation Bill Dement suggested me to study two groups of narcolepsy-cataplexy patients, placed on various daytime sleep schedules, to investigate the competition between the two types of sleep [3]. In addition, since we were dealing with participants in whom we could expect REM and NREM sleep-onset periods, we decided to study whether REM sleep and NREM sleep were equally able to relieve sleepiness. Finally, on a very practical basis, we took advantage of various daytime schedules, both in narcoleptics and controls, to determine whether narcoleptic patients can benefit from scheduled short naps [2]. Eighteen narcoleptic patients with both sleep attacks and cataplexy participated in the study as well as five normal controls. All participants were asked to stay in the sleep lab for a period of 58 hours including an adaptation night and two experimental 24-hour periods, referred to as days 1 and 2. Group 1 was permitted to sleep ad libitum on day 1. After each spontaneous awakening participants had to complete a self-rating scale on the restorative quality of their sleep and to perform a Wilkinson addition test. On day 2, participants were permitted to sleep at any time, but they were awakened 10 minutes after sleep-onset regardless of the type and stage of sleep. As on day 1, after each awakening, participants completed the self-rating scale and the Wilkinson addition test. Group 2 was placed on a fixed schedule of five testing sessions beginning at 09:00 am, 11:45 am, 02:00 pm, 04:15 pm, and 06:30 pm. Each session consisted of two 30-minute tests separated by a 15-minute break. An abridged form of the Wilkinson Addition Test was given in the first, third, and fifth sessions. A simplified serial counting test, the Serial Alternative Task was given in the second and fourth sessions. In addition to these tests, participants in group 2 had their sleepiness and mood quantified by means of two self-rating scales, the Stanford Sleepiness Scale and the Naval Psychological Research Unit Mood Scale. During day 1 the participants were not allowed to sleep between 7:00 am and 10:30 pm. If they fell asleep they were awakened after one minute of sleep. On day 2, the participants were asked to go to bed during each break, with the light turned off and the door closed. These were the only periods when the participants were permitted to sleep during the daytime. As concerns the competition between the two types of sleep, the mean sleep latency of sleep-onsets after lights out (group 2, day 2) emphasized the propensity of these patients to fall asleep, but the important point was the short latency of sleep-onsets whatever the kind of sleep onset. The mean sleep latency was even shorter for sleep-onset NREM periods (1 minute 33 seconds) than for sleep-onset REM periods (2 minutes 9 seconds). Thus this study offered further evidence that narcoleptic sleep attacks are probably both REM and NREM sleep in types. With regards to the recuperative value of sleep, the mean scale value of the participants’ ratings of the restorative quality of sleep was significantly higher when patients were allowed to sleep ad libitum than when they had their sleep interrupted after 10 minutes of sleep. Conversely, the different kinds of sleep made no difference at all. Finally, our results showed that 15-minute naps allowed narcoleptic patients to achieve a level of alertness in between their naps which was similar or better than that of normal controls under similar conditions..

#### The HLA story.

A number of studies in the past had shown a variable rate of probands with a positive family history of narcolepsy. In these studies, the suggested mode of inheritance was either autosomal dominant with an incomplete penetrance or multifactorial including genetic as well as environmental factors. These data prompted Yukata Honda to investigate HLA antigens in Japanese narcoleptic patients. I was not aware of that, but one day, in May 1982, as we were walking together on the beach of Milano Maritima, on the Adriatic Sea, Italy, Yukata told me mysteriously that he was making the diagnosis of narcolepsy « in the blood ». Thus when I returned to Montpellier, I started accumulating samples of blood from narcoleptic patients in view of HLA analysis. My friend, Jean Seignalet, an HLA specialist at the Institute of Hematology, Montpellier, was dubious about the interest of such an analysis, but he accepted the challenge. Meanwhile, Yukata Honda published an article revealing a significantly increased frequency of Bw35 and a significantly decreased frequency of Bw52 in 50 narcoleptic patients [4]. Sometimes later, Jean Seignalet called me and told me that there was something of interest in the analyses. Indeed he found a significantly increased frequency of B7 and a non-significantly increased frequency of A3 (an antigen in linkage disequilibrium with B7) in 38 French narcoleptic patients [5]. This discrepancy between Honda’s results and ours was not surprising as associations between HLA and diseases most often differ between caucasoid and mongoloid participants. Eventually and most importantly, Juji et al. found a 100% association between narcolepsy and HLA DR2 in 40 Japanese narcoleptic patients [6]. Later on, Langdon et al found a 100% association between narcolepsy and HLA-DR2-DQw1 in 37 English narcoleptic patients [7] and, in our turn, a 100% association between narcolepsy and HLA DR2-DQw1 in 23 French narcoleptic patients [8]. We were number 3!

#### Family studies in narcolepsy.

We were interested in further refining the proportion of participants with a family history of narcolepsy [9]. Out of a population of 188 unrelated narcoleptic probands, we identified 14 probands (7.44%) with a family history of narcolepsy, 23 (12.23%) with a family history of isolated repeated episodes of naps and or lapses into sleep, and 151 (80.31%) without a family history of either condition. Empirical risk for narcolepsy was 40.7% times greater among first-degree relatives of narcoleptics than in the general population. Narcolepsy and the condition characterized by isolated repeated episodes of naps and/or lapses into sleep have a common genetic component.

#### Modafinil.

Modafinil is a non-amphetamine central nervous system stimulant with wakefulness-promoting properties developed by Masson Ltd, a pharmaceutical company established in Maisons-Alfort near Paris. In 1974, two chemists, Assous and Gombert, identified a new molecule, adrafinil, later passed on to two pharmacologists, Duteil and Rambert, who observed that mice treated with this molecule were hyperactive. Two years later, the kinetic of adrafinil led to the identification of an active metabolite, modafinil. Modafinil went through several steps of development leading to the demonstration of a dose-dependent increase in locomotor activity in mice [10] and an increase in wakefulness and a decrease in sleep in the cat [11]. As soon as early 1983, Jouvet prescribed modafinil to narcoleptic patients and the results outdid the expectations. In 1984, Lafon Ltd decided to start clinical trials in both healthy volunteers and in narcoleptic and idiopathic hypersomnia patients which confirmed previous results [12]. Eventually, we conducted the first multicenter, randomized, placebo-controlled trial of modafinil, in fifty narcoleptic patients [13]. Modafinil was administered in a double-blind cross-over design, at a dosage of 300 mg versus placebo, and results were judged through questionnaires on therapeutic effects, sleep logs, polysomnography, and maintenance of wakefulness tests. An overall clinical benefit was noted by physicians as well as by patients. Above all, there was a significant improvement in the results of the maintenance of wakefulness tests for patients on modafinil in comparison with placebo (*p* < .05).

#### Idiopathic hypersomnia.

Idiopathic hypersomnia was for a long time confused with narcolepsy. It was only with the advent of polysomnography that the distinction between the two sleep disorders began to appear. Dement et al. were the first to suggest that participants affected by excessive daytime sleepiness without cataplexy, sleep paralysis, and sleep onset REM periods, should not be considered as having narcolepsy [14]. Later on, various terms were proposed to designate this entity and Roth was finally the first one to provide the clearest description of hypersomnia with sleep drunkenness [15] and to coin the term idiopathic hypersomnia [16]. Roth described two forms of this hypersomnia: a typical polysymptomatic form in which daytime sleepiness is accompanied by prolonged nocturnal sleep and usually by awakening difficulties and a monosymptomatic form marked solely by excessive daytime sleepiness with long naps. Three years after Roth’s description of idiopathic hypersomnia with two forms, the « Diagnostic Classification of Sleep and Arousal Disorders » [1] used the term « idiopathic CNS » and merged the two forms. In 1990, the « International Classification of Sleep Disorders » (ICSD) [17] came back to the term idiopathic hypersomnia and recognized alike a single form of the disorder with a prolonged major sleep episode and excessive daytime sleepiness consisting of prolonged (1 to 2 hours) episodes of sleep. Narcolepsy and idiopathic hypersomnia were included in a subgroup of dyssomnias, and the clinical significance of hypersomnia as an independent clinical entity was questioned. In 1997, we suggested to return to Roth’s initial distinction of a polysymptomatic and a monosymptomatic form [18]. In 2002, a revision of the ICSD was initiated by the board of directors of the American Academy of Sleep Medicine and a Task Force on hypersomnia of central origin was appointed, under the leadership of Emmanuel Mignot. Eventually, narcolepsy was distinguished into two different conditions, narcolepsy with cataplexy (NwC) and narcolepsy without cataplexy (NwoC) and idiopathic hypersomnia into two entities, idiopathic hypersomnia with long sleep time (IHwLST) and idiopathic hypersomnia without long sleep time (IHwoLST) [19]. However, there were still a number of pending issues including the distinction between IHwLST and IHwoLST challenged by the absence of clinical symptoms specific to one subgroup [20, 21] and the limits of the MSLT in differentiating the two conditions [22]. Hence, sustained efforts in the last few years to identify better narcolepsy type 1, narcolepsy type 2, IHwLST, and IHwoLST. One of these efforts was our data-driven cluster analysis based on clinical, polysomnographic, and MSLT variables in a population of 96 patients diagnosed with Nwc (*n* = 23), NwoC (*n* = 22), IHwLST (*n* = 26), and IHwoLST (*n* = 2) [23]. This study led to the identification of three main clusters of central hypersomnia, namely cluster1/combined IHwoLST/narcolepsy type 2 (*n* = 44); cluster 2: IHwLST (*n* = 27), and cluster 3: narcolepsy type 1 (*n* = 23). However, due to the absence of biological markers and robust electrophysiological tests, definitive diagnostic criteria for idiopathic hypersomnia are not yet available. Thus, we recently proposed a body of clinical symptoms, relevant scales and questionnaires, electrophysiological tests of various weighted sensitivity and specificity, and circadian control tests, for a rather reliable diagnosis of idiopathic hypersomnia and of its subtypes, IHwLST and IHwoLST [24].

#### Kleine-Levin syndrome.

My interest in Kleine-Levin syndrome was heralded by the observation and follow-up of a 13-year-old girl, while I was in the Stanford sleep clinic. This patient was affected with a rare form of Kleine-Levin syndrome referred to as menstruation-linked periodic hypersomnia [25]. Since that time I explored different aspects of the Kleine-Levin syndrome: Kleine-Levin syndrome in a 14-year-old girl: CSF hypocretin-1 measurements [26], Monozygotic twins affected with Kleine-Levin syndrome [27], Recurrent hypersomnia following traumatic brain injury [28] and a review based on the largest sample of case reports (339) ever reported [29], hence a rather comprehensive knowledge of the syndrome.

## Sleep Education

### Oral education

Teaching sleep physiology and sleep disorders was progressively added to the program of medical studies at the Montpellier Medical School. In the same spirit, at the request of general physicians, post-graduate education was developed in Montpellier and its province. This was a first step but we thought it necessary to establish higher education. In September 1987 we created a Montpellier University Diploma « Sleep and Wakefulness » for physicians and psychologists interested in sleep. It was a great success and 2 years later the Montpellier University Diploma was replaced by a French Inter-University Diploma. At a still higher level, all physicians and psychologists of our unit attended international meetings and symposia, either as simple participants or as speakers. Personally, I made presentations at annual sleep congresses and was invited to give lectures in almost 50 different countries located in Western and Eastern Europe, North, Central and Latin America, North Africa, Middle East, and Asia.

### Journals and books

« Sleep », the first journal in the field of sleep research came out in the fall of 1977, with William C. Dement and Christian Guilleminault as founding editors and Christian Guilleminault as editor-in-chief. «Journal of Sleep Research », the official journal of the European Sleep Research Society (SRS) was launched in 1992 with Jim Horne as the editor-in-chief. « Sleep Medicine Reviews » was established in 1992, with Michael Vitiello and Jean Krieger as editors-in-chief. « Sleep Medicine » was born in 2000 with Sudhansu Chokrovrty as the editor-in-chief. In France « Médecine du sommeil » was launched in 2004 with myself as the editorial coordinator and « The Journal of Clinical Sleep Medicine » the official journal of the American Academy of Sleep was created in 2005 with Stuart F. Quan as editor-in-chief. In addition, a lot of books have been published for continuous education, in France as well as in other countries, and my personal input was the publication of 13 books, nine in French and four in English.

### Sleep societies

In 1961, a small group of American researchers founded what eventually became the SRS, while the European Society of Sleep Research (ESRS) was founded in 1972 with Werner P. Koella as its president. Personally, I had the chance to be a member of the European SRS since its foundation in 1972, and later a member of the SRS (USA) from 1974 on and in 1978–1979 a member at large of the same society. As regards the European SRS I was the secretary of the society from 1980 to 1984 under Ian Oswald’s presidency, a member of the scientific committee from 1986 to 1990, and its president from 1996 to 2000, together with Myriam Kerkhofs as vice-president, Domien Beersma as secretary, Igino Fagioli as assistant secretary and Jürgen Zulley as treasurer, a fascinating and time-consuming job.

### European Commission

Toward the end of my career, I had the chance to be appointed as an independent expert acting as a reviewer of the European program 507231 SENSATION, Advanced Sensor Development for Attention, Stress, Vigilance, and Sleep–Wakefulness Monitoring. Together with me were J.T. Devreese, professor emeritus of theoretical physics at the University of Antwerp, Belgium, and Pietr Grabiec, professor of mechanics at the Institute of Electron Technology, Warsaw, Poland. The task involved the review of multiple reports and the participation in meetings held in Reykjavik, 2004; Warsaw,2005; Louvain, 2006; Thessaloniki, 2007; and Stockholm, *2008*. This was a fascinating opportunity to meet and work with highly skilled specialists working in multiple fields.

## Future of the Sleep and Wake Disorders Center in Montpellier

Sleep medicine is still a fragile field in comparison with cardiology, neurology, pneumology, and others. Some sleep disorder centers have collapsed after their chairman’s retirement. Indeed, one of the most difficult tasks for the chairman of a medical, surgical, or biological department is to find a successor, equal to himself or preferably superior, and this is particularly the issue in sleep medicine, which is not yet a fully established specialty in most countries. Thus, I was lucky enough to have Yves Dauvilliers as an intern and a resident in our department. He accepted to take over from me. Yves has upgraded the level of research at the center. He has published more than 500 papers within the last 20 years. He is internationally known and a member of the French Academy of Medicine. Long live Yves and the Montpellier Sleep and Wake Disorders Center!

## Notes

This paper is part of the Living Legends in Sleep Research series, which is sponsored by Idorsia Pharmaceuticals and Jazz Pharmaceuticals.

## Appendix

**Appendix Table 1.** Precursors of Sleep Medicine in Europe (Before 1975)

Names of precursors	Affiliations	Main interest (s)	Approximative date of first PSG
Vera Dürrigl	EEG and Clinical Physiology Psychiatric Hospital Vrapce Zagreb, Croatia	Psychiatry and sleep	Early 1970’
Bedrich Roth, Sona Nevsimalova	Department of Neurology Faculty of Medicine Charles University, Prague Czechoslovakia	Hypersomnia’s of central origin	1965
Michel Jouvet, C. Fischer-Perroudon, Jacques Mouret	Sleep laboratory. Edouard Herriot Hospital, Lyon, France	Morvan’s fibrillar chorea	1962
Pierre Passouant, Michel Baldy-Moulinier, Marion Delange	Department of Clinical Neurophysiology St Charles Hospital Montpellier, France	Epilepsy and sleep. Narcolepsy Sleep in the new born	1961
Colette Dreyfus-Brisac, Nicole Monod	Center for Neonatal Biological Research, Port-Royal Hospital Paris, France	Neonatal medicine	1962
Daniel Kurz	Department of Clinical Neurophysiology University Hospitals, Srasbourg, France	Sleep disorders breathing	1970
Odile Benoît, Lucile Garma, Françoise Goldenberg	Department of Clinical Neurophysiology Pitié-Salpêtrière Hospital Paris, France	Parasomnias, insomnia	1971
Uros J Jovanovic	Department of Neurology, University Hospital, Würzburg, Germany	Neurology and sleep, Psychiatry and sleep, Sexual disturbances and sleep	1964
Hartmut Schulz	Max Plant Institute für Psychiatry Munich, Germany	Sleep structure, sleep and depression	1972
Elio Lugaresi, Giorgio Coccagna	Institute of Neurology, University of Bologna, Bologna, Italy	Sleep related movements, Sleep disorders breathing, parasomnias	1965
Salvatore Smirne	Department of Neurology, Institute San Raffaelo,Milan, Italy	Insomnia, parasomnias, Sleep disorders breathing	1969
Alberto Muratorio	Institute of Neurology Universith of Pisa, Pisa, Italy	Depression and sleep, Neurological diseases and sleep	1969
Drzej Jus and Karolina Jus	Department of Psychiatry, Medical University, Warsaw, Poland	Mental disorders and sleep, Psychotropic drugs and sleep	1966
Julius Narebski	Department of Pediatric Neurology Torun and Bydgoszcz, Poland	Sleep in children	1972
Liviu Popoviciu	Department of Neurology. Tirgu-Mures Hospital, Tirgu-Mures, Rumania	Hypersomnia of central origin	1971
Ian Oswald, Vlasta Brezinova	Department of Psychological Medicine, Royal Edinburgh Hospital, Edinburgh, Scotland, UK	Insomnia, pharmacology of sleep	1959
Antonio Vela-Bueno, Rosa Peraita-Adrados	Department of Clinical Neurophysiology Hospital San Carlos, Madrid, Spain	Sleep and Epilepsy, Full spectrum of sleep disorders	1971
Teresa Sagales, Dolores de la Calzada	Department of Clinical Neurophysiology Hospital Val d’Hebron Barcelona, Spain	Sleep disorders breathing, Neurological disorders and sleep	1972
Antonio Beneto	Department of Neurophysiology, University Hospital La Fe, Valencia, Spain	Neurological disorders and sleep	1972
Jean-Michel Gaillard	EEG Laboratory, University Hospital Chêne-Bourg, Geneva, Switzerland	Insomnia, pharmacology of sleep, Quantitative analysis of sleep	1969
Alexsandr Vein	Depart. of Neurology, Sechenov first Moscow Medical Institute, Moscow, USSR	Neurological disorders and sleep, Insomnia	1968
